# Ageing with and without HIV: will advanced age bring equity or greater disparity?

**DOI:** 10.1002/jia2.25400

**Published:** 2019-10-01

**Authors:** Amy C Justice, Janet P Tate

**Affiliations:** ^1^ Veterans Affairs Connecticut Healthcare System West Haven CT USA; ^2^ School of Medicine Yale University New Haven CT USA; ^3^ School of Public Health Yale University New Haven CT USA

**Keywords:** middle age, old age, HIV, cancer, extended care, end‐of‐life

Our understanding of the long‐term complications of HIV infection and their interaction with ageing and treatment evolves as the bulk of the population of people living with HIV ages. We have a reasonable understanding of what living with HIV means for middle aged (50 to 64 years) men in upper income countries. We are only beginning to grasp how ageing with HIV plays out for women 1, for individuals in mid to lower income countries 2, or for those who initiated more optimal antiretroviral treatment. Finally, we are only beginning to understand how long‐term HIV infection may interact with what gerontologists consider “older age” (65+ years).

The evolution of our understanding of ageing with HIV began after sulfamethoxazole and trimethoprim (Bactrim) prophylaxis combined with AZT modestly improved survival and we recognized HIV‐associated neurocognitive disorder (HAND). Once double and triple combination therapy became widespread, we began seeing higher rates of cancer, cardiovascular disease, renal insufficiency and liver disease among people living with HIV (PLHIV). First attributed to treatment toxicity, these conditions were later recognized as longer‐term complications of HIV 3. At the same time, rates of HAND decreased.

We also began to talk about “accelerated ageing” and “frailty,” but these concepts may have been a bit exaggerated 4, 5 in a largely middle‐aged, ambulatory population with suppressed HIV‐1 RNA who were living independently. Once differences in the underlying distribution of ages in the population with HIV versus the general population and higher rates of smoking are accounted for, people ageing with HIV do not appear to develop specific age‐associated conditions such as cancer, cardiovascular disease or renal disease at substantially earlier ages than the general population. Instead, they have a modest increased or accentuated risk across age groups. Furthermore, pre‐frailty is much more common than frailty, especially in this middle‐aged population.

An important theme in ageing is that no two people age in the same way. With advanced age, the most salient issue is not the number of years one has lived, but the cumulative “slings and arrows of outrageous fortune” that they have experienced in their lives, including multimorbidity and polypharmacy 6. For example, compared to younger individuals, prognostic indices for risk of mortality may be less accurate among those of more advanced age 7 and most frailty metrics such as those developed by Fried 8 and Rockwood 9 omit age, even though age often remains an independent predictor of frailty related outcomes 10.

We may see growing disparities in health outcomes among those ageing with HIV as they reach more advanced ages. Those who achieved viral load suppression prior to experiencing substantial CD4 loss, who either stop or never start smoking or drinking harmful amounts of alcohol, those who avoid excessive weight and who are physically active will experience “successful ageing” 11, 12, 13. Those who did not receive early treatment, who start or continue to use substances, those who remain or become overweight or obese, and those who fail to stay active, will experience poorer health related quality of life with excess morbidity and mortality compared with uninfected individuals.

Nevertheless, while the incidence of major comorbid diseases demonstrates variable associations with age, risks are typically accentuated with advancing age and often in a nonlinear manner. When PLHIV are compared with age‐matched uninfected individuals, their excess risk for anal, lung, liver and oral cavity/pharyngeal cancers increases after 50 years of age 14. Furthermore, the association with advancing age is nonlinear. For example, consider recent SEER data on cancer incidence by age in the general population (Figure 1). Risk of colon cancer, lung cancer, prostate cancer and Non‐Hodgkin lymphoma cancer demonstrate a roughly exponential association with age. Compared to those aged 50 to 59, those aged 70 to 79 have an approximately fourfold risk of colon cancer, prostate cancer, and Non‐Hodgkin lymphoma and a sixfold risk of lung cancer. Relative risk increases more modestly for anal cancer, liver cancer, breast cancer and Hodgkin lymphoma. Increased relative risks after 50 years of age suggest that the excess risk we have observed among a predominantly middle‐aged population of PLHIV compared to age‐matched uninfected individuals may be amplified in the future 6. Compared with uninfected individuals of the same age, we may begin to see larger disparities in clinical outcomes for PLHIV with advancing age, a phenomenon known as accentuated rather than accelerated ageing.

**Figure 1:**
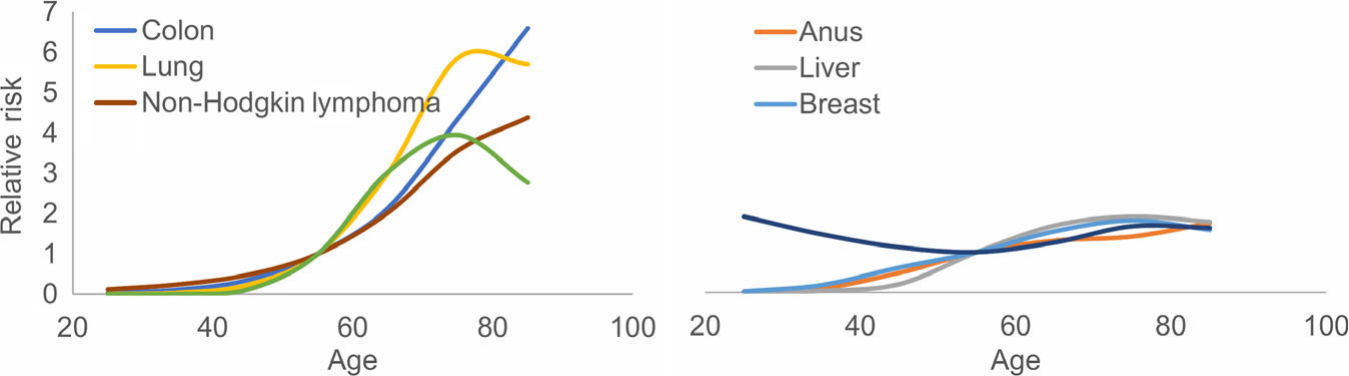
Relative risk of selected cancers compared to age 50 to 59 years in the general US population. From Surveillance, Epidemiology and End Results (SEER) Programme. Colon does not include rectum, lung includes bronchus, anus includes rectum, breast is female only, prostate is male only.

While we have made miraculous strides in extending life and quality of life with HIV infection 15, 16, let us not forget that successful ageing includes effective symptom palliation throughout the ageing process and appropriate accommodations at the end‐of‐life. Symptom palliation requires careful assessment of 17, 18, and focused efforts to manage, bothersome symptoms 19 without exacerbating the problem of polypharmacy 20. Providers must also help patients and patients loved ones recognize when a transition from independent living to supported care and end‐of‐life planning are indicated. Nursing homes and other extended care facilities and home care programmes will need to integrate increasing numbers of PLHIV into their care, with appropriate expertise and without stigma 21, 22. These decisions will require thoughtful discussion with patients and their loved ones. Timing of transitions in care and end‐of‐life planning might be better informed by comprehensive prognostic models such as the Veterans Aging Cohort Study Index (VACS Index) 23.

Ageing with HIV will likely remain somewhat different from ageing without HIV, but much can be learned from the geriatric literature regarding deprescribing, nonpharmacologic symptom management, transitions in care and end‐of‐life planning. We do not yet know what the next several decades will hold, but it is likely that we will see increased cancer incidence, greater numbers of extended care admissions, and, eventually, higher age‐related mortality. Care providers and clinical researchers will need to adapt what has been learned in other ageing populations to PLHIV as they age beyond 65 and 70 years.

## Competing interests

No conflict.

## Authors’ contributions

ACJ conceived of the idea for this editorial and wrote the first draft.

JT reviewed the draft, edited the scientific content and constructed the figure.
